# Pediatric cardiac services in Sudan: Achievements, challenges, and future perspectives (2004–2021)

**DOI:** 10.3389/fped.2022.793188

**Published:** 2022-11-09

**Authors:** Sulafa Ali, Mohamed Eamin A.M.E. Medani

**Affiliations:** ^1^ Consultant Pediatric Cardiologist, Sudan Heart Center, University of Khartoum, Khartoum, Sudan; ^2^ Consultant Pediatric Cardiologist, Wad Medani Heart Center, University of Gazira, Wad Medani, Sudan

**Keywords:** pediatric, cardiac, surgery, Sudan, cardiac catherization

## Abstract

Pediatric cardiology (PC) is a rapidly advancing specialty addressing a large population of children as well as adults with congenital heart disease. It requires huge technical and financial resources; therefore, establishing, maintaining, and developing such services in limited resource settings are challenging. A PC program that includes clinical aspects, echocardiography, diagnostic and interventional cardiac catheterization and cardiac surgery, and rheumatic heart disease control was established in Sudan in 2004. There are currently three public centers with facilities to evaluate and treat children with heart disease: two in Khartoum and one in Wad Medani. Major obstacles include the shortage of trained personnel and operation rooms, the deficiency of intensive care facilities, and the financial burden of interventional procedures. This paper details the establishment and progress of the program, its challenges, potential solutions, and future perspectives for PC programs in Sudan and African countries.

## Introduction

Congenital heart disease (CHD) is considered the most common form of congenital malformation affecting about 1% of live birth with variable prevalence across countries. In resource-limited settings, accurate prevalence rates might not be readily measurable; however, due to the high birth rates, CHD is expected to have at least a similar, if not greater, prevalence than developed countries (1). It is estimated that almost 90% of patients in low- and middle-income counties have limited access to cardiac care (2).

There have been huge advances in the diagnosis and management of CHD in the last decades including cardiac imaging, cardiac catheterization (Ccath) interventions, and refinements of surgical techniques and postoperative care, allowing most children with CHD to live up to adulthood. Such advanced treatment needs expensive and technically demanding settings that can challenge resource-limited health systems.

In addition to CHD, rheumatic heart disease (RHD) is endemic in many developing countries, leading to the premature death of children and young adults. The echocardiographic (echo) prevalence of RHD was reported to be up to 26% (3). Although it is entirely preventable, it is imposing a significant health and economic burden on developing countries, particularly in Africa.

Pediatric cardiology (PC) services started in Sudan in the 1980s; however, the services were sustained only for a few years, mainly due to a shortage of trained staff (4). In 2002, noninvasive PC and CHD surgery were introduced at the Sudan Hear Center (SHC), and in 2004, a Department of PC was established, which acted as the nucleus for the current PC program (5).

This review highlights the achievements and challenges of PC services in Sudan and the future perspectives of PC programs in Africa.

## Establishing the pediatric cardiology program at Sudan Hear Center (2004–2011)

**Figure 1:**
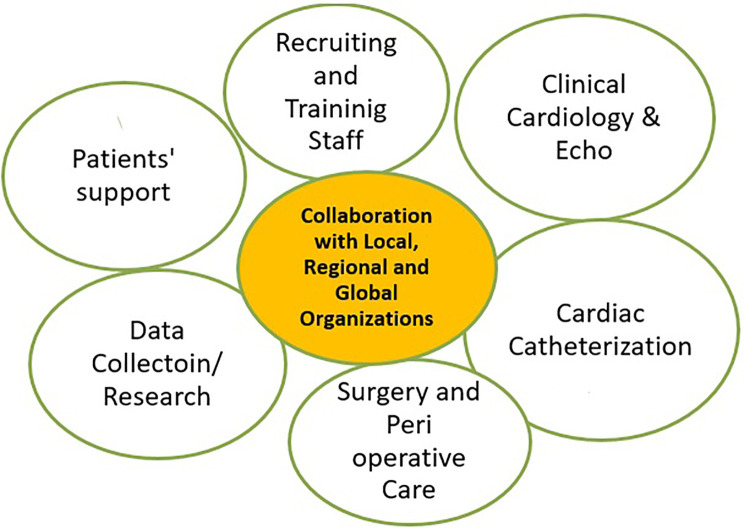
Establishment of the Department of PC at SHC, 2004–2011. PC, pediatric cardiology; SHC, Sudan Hear Center.

A multidisciplinary PC program was established at the SHC in 2004 that consists of the following activities as depicted in Figure 1.

### Recruiting and training of staff

The Department of PC at SHC started by recruiting residents and specialists with an interest in cardiology and setting educational and service meetings. Training in clinical cardiology and echo was conducted through hands-on sessions, lectures, and workshops. A 24-h-a-day on-call system was initiated to cover consultations and emergencies. In 2006, trained specialists were able to run two more clinics at the SHC and two satellite clinics at the main Children's Hospital. Two candidates were sent to Malaysia for training in cardiac intensive care and Ccath.

### Echocardiography

Until 2004, echo was mostly performed by adult cardiologists and the echo reports were not standardized. The PC program introduced the standard segmental approach; echo manuals with reference values were provided to PC clinics. Videos and, later on, digital recordings of echo studies assisted the discussion of patients with surgeons and the training of cardiologists (6).

### Cardiac catheterization

**Figure 2:**
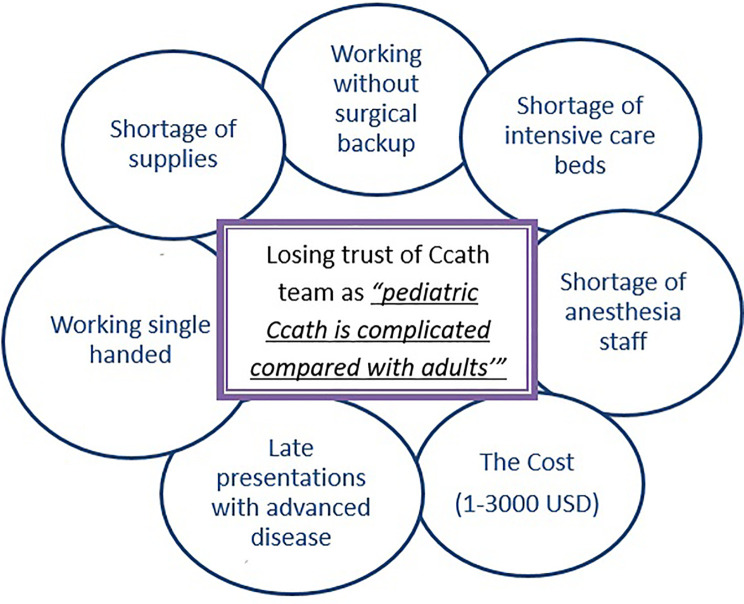
Challenges that Ccath faced in the establishment phase in 2004–2011. Ccath, cardiac catheterization.

Pediatric Ccath procedures started in 2004 with simple diagnostic and interventional cases. The program faced many challenges, as shown in Figure 2. The cases were diverse, complex, and commonly present in advanced stages of diseases, adding to the risk for the patients and the difficulty for the single-handed operator.

Despite difficulties, the program slowly progressed, performing an average of 150 cases per year, including an increasing number of interventional procedures. Currently, we perform routing device closures, balloon dilatation, and emergency procedures.

### Surgery and perioperative care

Surgery for CHD requires a multidisciplinary team, including intraoperative and postoperative facilities. Postoperative resources including intensive care unit (ICU) equipment and supplies and medical and nursing staff availability were the most challenging aspects in our settings. In 2002–2006, a surgeon from abroad was hired by SHC and managed to operate on about 120 cases per year with good outcomes (7). In 2006, the first Sudanese CHD surgeon joined SHC and Ahmed Gasim Heart Center (AGHC). Postoperative care was conducted by the surgical staff assisted by the PC team, and there was no cardiac intensivist. Collaboration with the following organizations assisted the program during this period:

I. C*hildren's Heart Foundation (United States)* visited SHC in 2003, 2004, and 2005. In each visit, about 20 operations and Ccath procedures were performed. Surgery included all ranges of complexities, including Rastelli and arterial switch procedures.
II. In 2006, 2007, and 2008, a PC team from *Qatar* helped to establish interventional catheterization at SHC. In each visit, about 20 cases were completed, including device closure and balloon dilatation. These visits supported the training of our staff and paved the way for routine device closure in Sudan. Of note, these visits were totally free of charge and sponsored by the Qatar Red Crescent.
III. A team from *Kingdom of Saudi Arabia* visited biannually and performed Ccath and surgery on a large number of patients in SHC, AGHC, and Wad Medani Heart Center (WMHC). The team assisted the local program training in ICU and Ccath lab. This team still visits Sudan on a regular basis.

### Data collection and research

CHD registry using simple paper records was established and reported the clinical and echo features of CHD and cardiomyopathies in Sudanese children (8–10).

### Patients’ support: charity organizations’ contribution

A charity organization, Sudanese Children's Heart Society (SCHS) (www.sudankidsheart.org), was established to assist children and their families and provide service and training in remote areas. The society contributed to RHD control program implementation. Other organizations that assisted patients include SADAQAT, Patient Support Fund, and Friends of Heart Patients Association, among others.

An Italian Missionary Hospital led by *Emergency* Organization opened a cardiac center in 2007. The center is mainly performing valve surgery for patients with RHD, free of charge, and it helped many children with RHD from Sudan and nearby African countries.

## Establishment of pediatric cardiology and surgery at Ahmed Gasim Heart Center

From 2003 to 2016, two pediatric cardiologists and one surgeon established and maintained the services, with an average of two clinics and four to six surgeries per week.

### Establishment of pediatric cardiology at Wad Medani Heart Center

A pediatric cardiologist has established and been still running the services—single handedly—since 2010. The average number of cases seen in outpatients and inpatients sections is 3,300 per year. The average number of Ccath cases, performed by visiting teams, is 100 per year.

### Pediatric cardiology program development from 2012 to 2021

**Figure 3:**
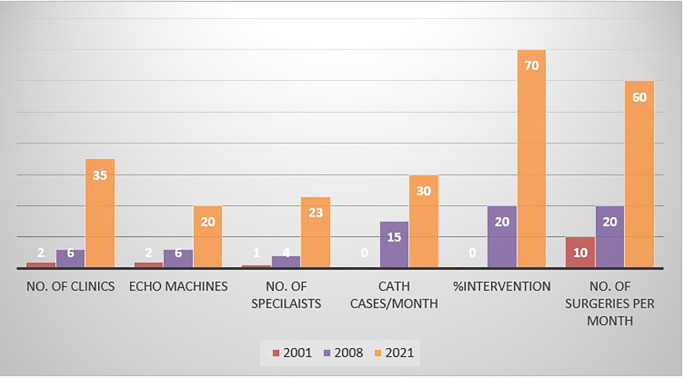
Growth of PC services in Sudan from 2001 to 2021. PC, pediatric cardiology.

During the last 9 years, there have been important advancements in PC services. The growth of PC service from 2001 to 2021 is shown in Figure 3.

#### Pediatric cardiology fellowship program

**Figure 4:**
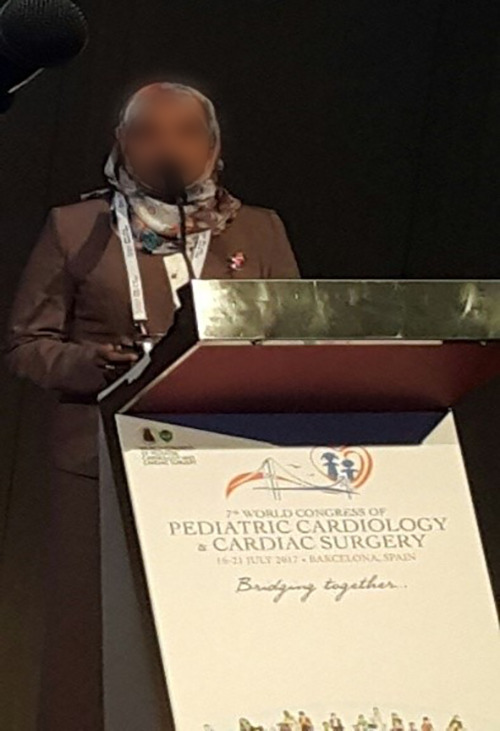
PC fellow (Dr Sara Domi) presenting at the World Conference of Pediatric Cardiology and Cardiac Surgery meeting in Barcelona, in 2017. PC, pediatric cardiology.

**Figure 5:**
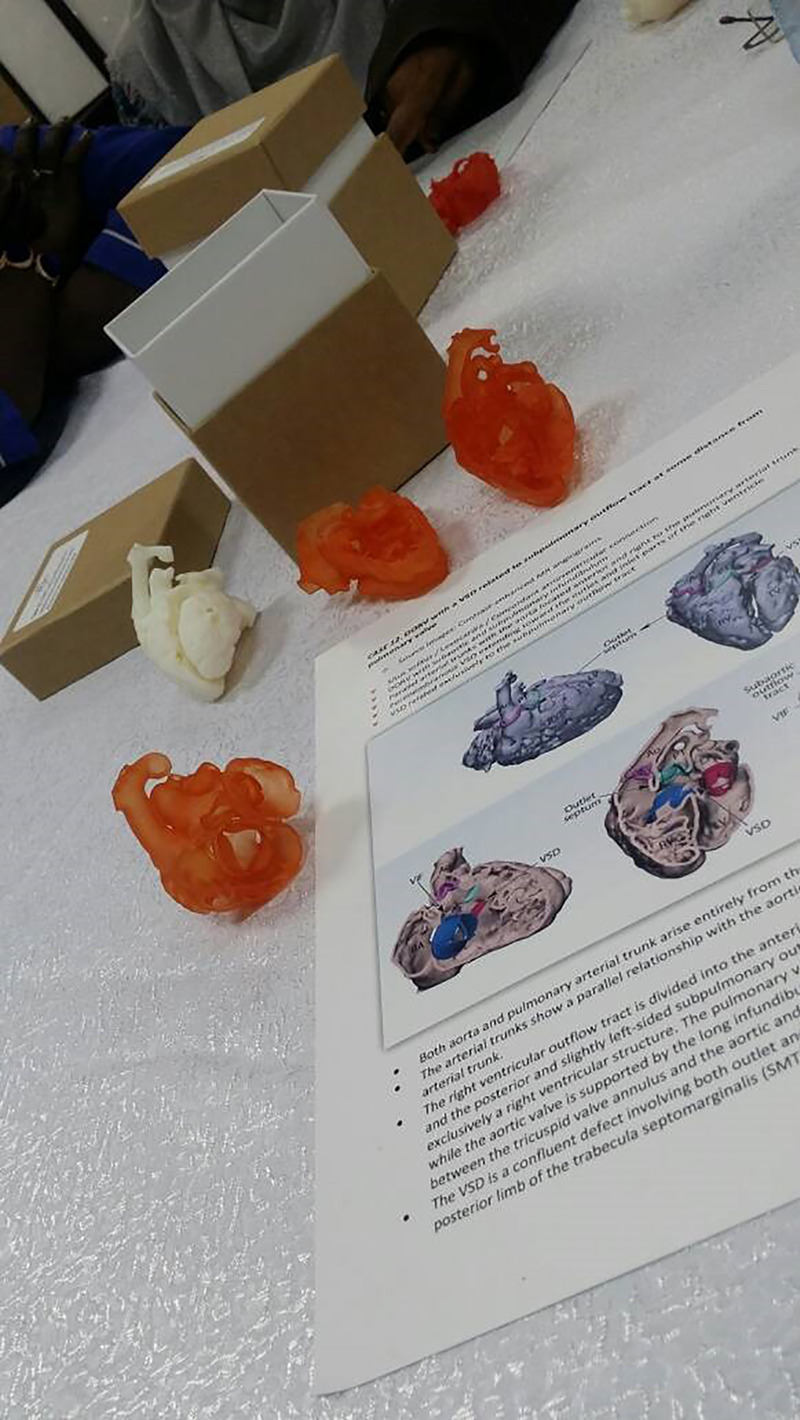
Ten sets of 3D printed hearts with double outlet right ventricles donated by Stollery Children's Hospital Team for the Sudan PC training program. PC, pediatric cardiology.

In 2012, a PC fellowship program was approved by the Sudan Medical Specialization Counsel. This is a 3-year program that includes training in clinical cardiology, echo, and Ccath. Candidates were involved in research activities and contributed to local and international conferences (Figure 4). To overcome the shortage of local trainers, speakers from abroad were invited to deliver 3–5-day courses and hands-on sessions. In addition, online teaching and evaluation sessions were increasingly utilized, particularly during the COVID pandemic. A team from Stollery Children's Hospital (Edmonton, Canada), who visited SHC for patient's management, conducted annual workshops and donated 10 sets of three-dimensionally printed hearts for fellow training (11) (Figure 5).

To date, 11 batches have been enrolled, 17 cardiologists have graduated, and 8 are in training. Of those who graduated, only eight are practicing in Sudan, while nine emigrated abroad. Despite that, the graduates made a significant impact on PC services. In addition to the fellowship program, a 6-month training period (focused training) was offered to selected candidates from rural Sudan who established two clinics in two remote districts.

#### Rheumatic heart disease control program

In collaboration with the Sudan Heart Society and the Ministry of Health, an RHD control program was initiated in 2012 (12). The program conducted echo screening, health worker training, and public awareness sessions in endemic areas.

#### Advancement of cardiac surgery and intensive care

Two more CHD surgeons joined the country, and the number and complexity of procedures improved over the last years, allowing for performing open heart surgery in infants weighing less than 5 kg. Two pediatric cardiac intensivists established dedicated pediatric cardiac ICUs, a crucial achievement that led to the improvement of surgical outcomes. Training of physicians, nurses, and other paramedical staff in the ICU has made a significant contribution to patients' postoperative care.

A cardiac surgery fellowship was established at the Sudan Medical Specialization Counsel. Training in general cardiothoracic surgery is followed by specialty training in congenital or adult surgery in collaboration with centers abroad.

#### Government commitment to funding of Ccath and surgery

From 2001 to 2018, the patients had to pay 50% of the cost of cardiac interventions, which was a major obstacle to the services. Starting in 2018, the government sponsored all Ccath procedures to be free of charge for all (adult and pediatric) patients. This has made a significant impact on patient care and improved the potential for training. In the year 2020, the government approved a plan for free-of-charge pediatric surgical operations expected to start in the year 2022. This will pave the way for increasing the number of trained staff and ICUs to cover the increasing demand for surgery.

## Challenges to pediatric cardiology services in Sudan

**Figure 6:**
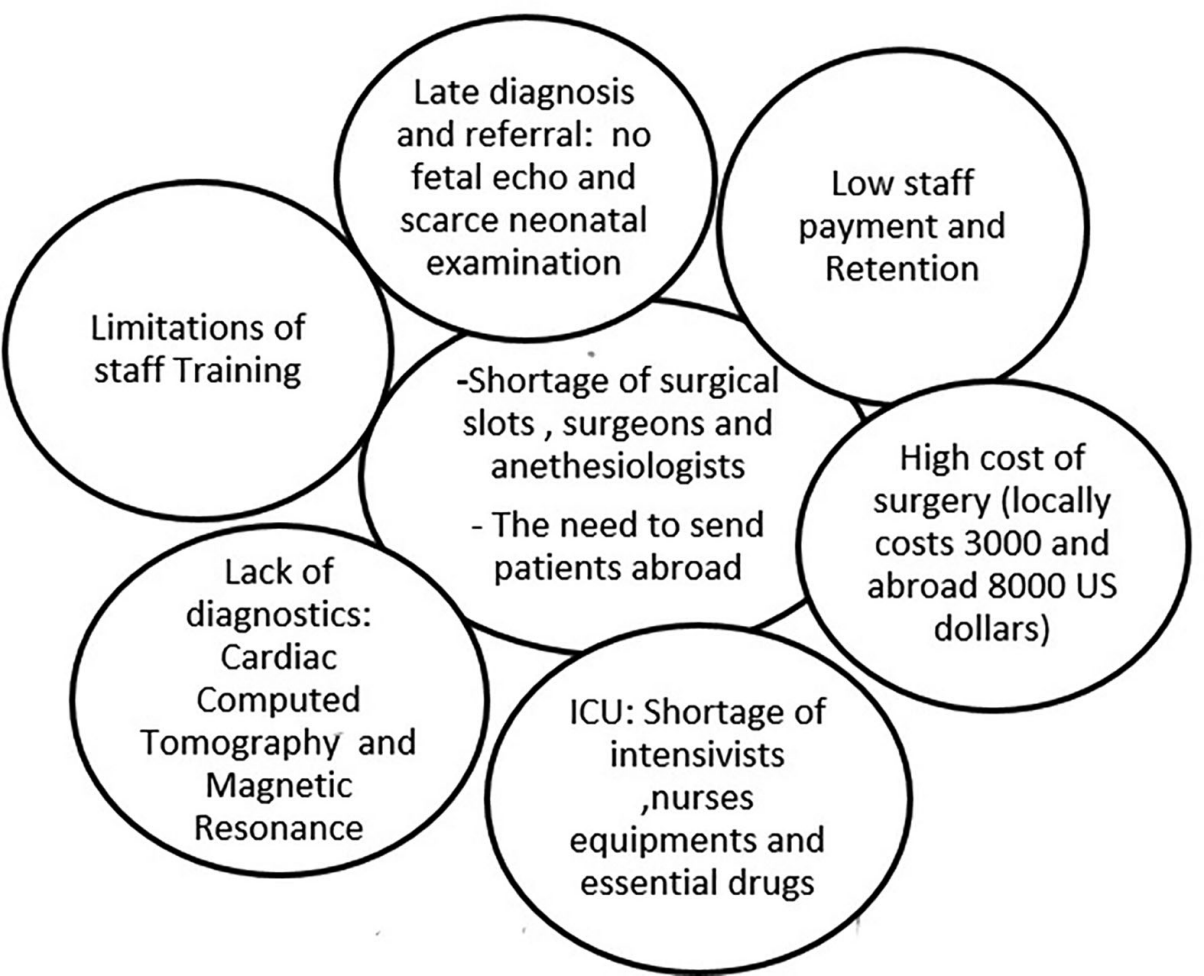
Challenges to PC services in Sudan. PC, pediatric cardiology.

The challenges that PC faces in Sudan are detailed in Figure 6.

## Pediatric cardiology programs in Africa

The situation of PC in African countries has many similarities to Sudan. Similar challenges are faced in eastern Africa, where only a few PC centers are available in Uganda, Kenya, and Tanzania (13). A similar experience has been reported in Ghana and Namibia, where services were gradually introduced but still lacked the necessary infrastructure and sustainable funding (14, 15).

Of particular note, accredited fellowship programs are scarce in Africa, which threatens the development and sustainability of PC programs. African–African communications are highly needed, which can be facilitated through organizations such as the Pan African Network of Pediatric and Congenital Heart Disease (PANPACH) (4).

South Africa's vast experience has been an asset to other African countries, particularly the sub-Saharan area (15). This includes both patient management and training in many pediatric subspecialties, including PC, which enabled many Africans to return and establish services in their home countries (16).

Another important link has been established between many African countries and Indian institutions with robust PC services. Indian centers accept many African patients for management and also contribute to training. However, the cost of intervention has been an important obstacle to the optimal utility of African/Indian collaboration. The links could be strengthened through institutional agreements for training rather than the current medical broker's management (17).

## Insights into future perspectives

**Figure 7:**
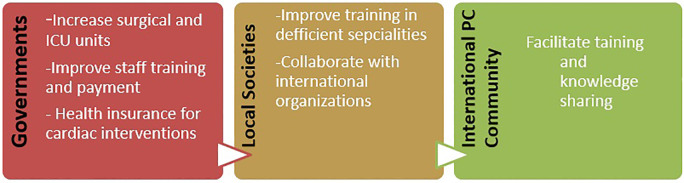
Governments' and local and international PC organizations' roles in consolidating PC services across developing countries. PC, pediatric cardiology.

Government commitment is needed to improve the number of surgical and ICU units, provide diagnostic tools such as cardiac computed tomography imaging, and improve staff payment and retention. In Sudan, as well as other developing countries, there is a need to introduce PC subspecialties such as fetal echo, electrophysiology, and adult CHD. At a later stage, more complex Ccath interventions and introducing neonatal open heart surgery will be possible after the full establishment of ICU facilities. Private sector involvement is encouraged and expected to decrease the number of patients needing treatment abroad; however, governmental and charitable organizations need to support needy families. Collaboration between local scientific societies with regional/international organizations such as the World Society for Pediatric and Congenital Heart Surgery is highly needed to create training opportunities either locally by invited trainers or abroad in regional centers of excellence (Figure 7).

## Conclusion

PC services in Sudan have been established and managed to grow despite challenges. Like many other developing countries, the main obstacles are a shortage of trained staff, high cost of procedures, and deficiency of operation rooms and ICU facilities. Sudan managed to establish a fellowship program that assisted the sustainability and progress of PC services. Collaboration between local and regional/international organizations is important in bridging the vast gaps in PC services in Sudan and similarly low-income countries.
